# Tumor PKCδ instigates immune exclusion in EGFR-mutated non–small cell lung cancer

**DOI:** 10.1186/s12916-022-02670-0

**Published:** 2022-12-08

**Authors:** Yi-Han Zuo, Wei-Na Gao, Ya-Jia Xie, Sheng-Yong Yang, Jin-Tai Zhou, Hai-Hai Liang, Xing-Xing Fan

**Affiliations:** 1 Dr. Neher’s Biophysics Laboratory for Innovative Drug Discovery, State Key Laboratory of Quality Research in Chinese Medicine, Macau University of Science and Technology, Macau, China; 2 Department of Cardiology, Harvard Medical School, Boston, MA USA; 3 Department of Chemistry, Southern University of Science and Technology, Shenzhen, Guangdong China; 4 State Key Laboratory of Biotherapy and Cancer Center, West China Hospital, West China Medical School, Sichuan University, Chengdu, China; 5 TianJin Medical University General Hospital, Tianjin, China; 6 Department of Pharmacology, College of Pharmacy, Harbin Medical University, Harbin, Heilongjiang China

**Keywords:** Tumor microenvironment, Immune checkpoint, Tumor infiltrating lymphocytes, PKCδ, PD-1

## Abstract

**Background:**

The recruitment of a sufficient number of immune cells to induce an inflamed tumor microenvironment (TME) is a prerequisite for effective response to cancer immunotherapy. The immunological phenotypes in the TME of EGFR–mutated lung cancer were characterized as non-inflamed, for which immunotherapy is largely ineffective.

**Methods:**

Global proteomic and phosphoproteomic data from lung cancer tissues were analyzed aiming to map proteins related to non-inflamed TME. The ex vivo and in vivo studies were carried out to evaluate the anti-tumor effect. Proteomics was applied to identify the potential target and signaling pathways. CRISPR-Cas9 was used to knock out target genes. The changes of immune cells were monitored by flow cytometry. The correlation between PKCδ and PD-L1 was verified by clinical samples.

**Results:**

We proposed that PKCδ, a gatekeeper of immune homeostasis with kinase activity, is responsible for the un-inflamed phenotype in EGFR-mutated lung tumors. It promotes tumor progression by stimulating extracellular matrix (ECM) and PD-L1 expression which leads to immune exclusion and assists cancer cell escape from T cell surveillance. Ablation of PKCδ enhances the intratumoral penetration of T cells and suppresses the growth of tumors. Furthermore, blocking PKCδ significantly sensitizes the tumor to immune checkpoint blockade (ICB) therapy (αPD-1) in vitro and in vivo model.

**Conclusions:**

These findings revealed that PKCδ is a critical switch to induce inflamed tumors and consequently enhances the efficacy of ICB therapy in EGFR-mutated lung cancer. This opens a new avenue for applying immunotherapy against recalcitrant tumors.

**Graphical Abstract:**

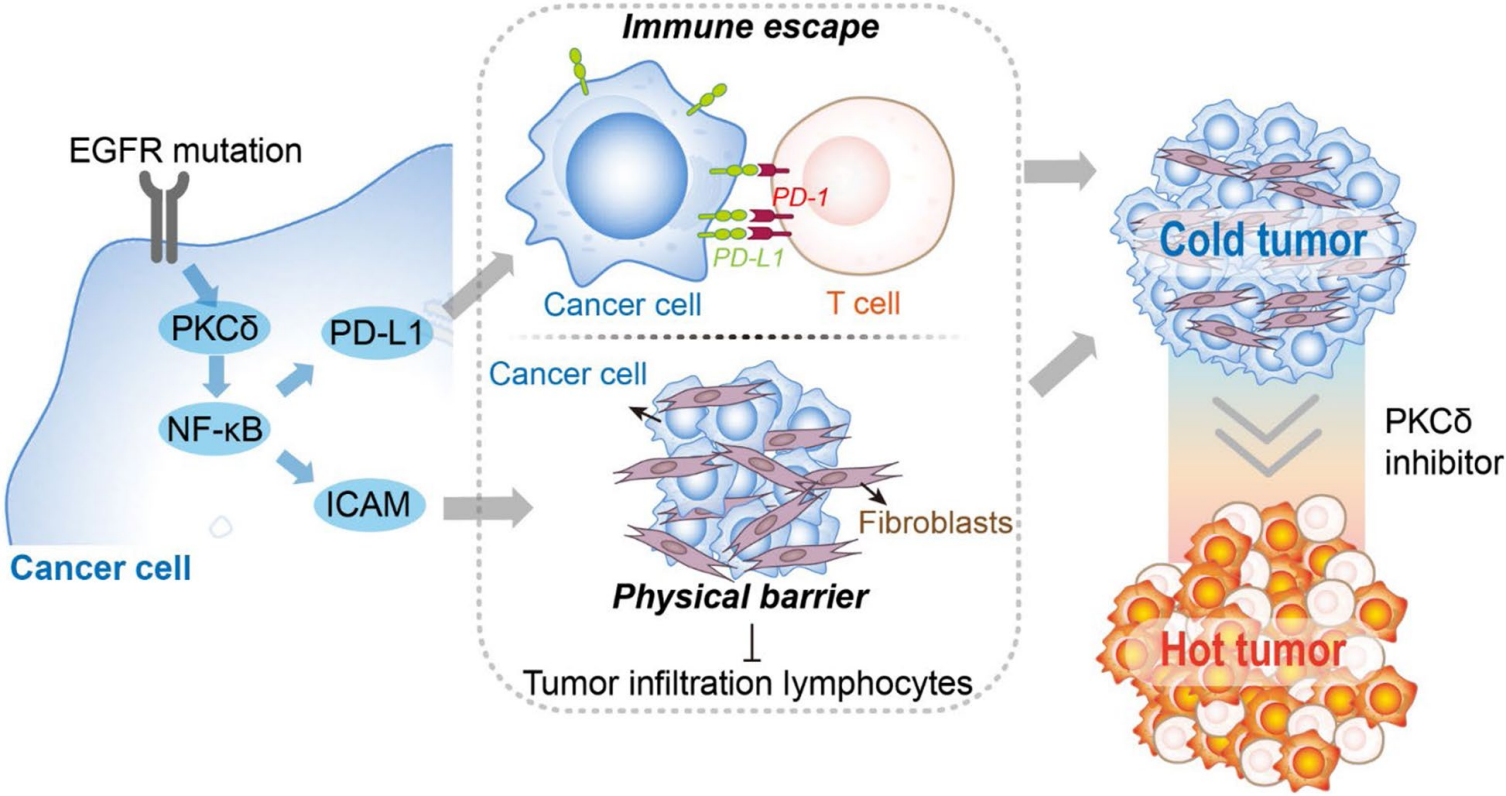

**Supplementary Information:**

The online version contains supplementary material available at 10.1186/s12916-022-02670-0.

## Background

Immune checkpoint blockades (ICBs) therapy opens a new era of cancer immunotherapy, which creates a holistic version and radically changes for cancer treatment [1, 2]. Unfortunately, its clinical application has been limited by a low response rate. Especially, certain molecular subgroups of cancer, like EGFR-mutated lung cancer, were reported to obtain low efficacy of ICB therapy in clinical [3, 4]. EGFR mutation leads to tumor immune escape and compromises infiltration of tumoricidal effector of T cells [5]. Although tumor microenvironment (TME) restriction on immune cells has been well studied [6–8] and significant efforts have been taken to explore the potential way to enhance T cell infiltration into the tumor bed [9, 10], the mechanism of EGFR-mutation inducing un-inflamed TME remains unknown. With the aim of mapping proteins related to the non-inflamed TME of EGFR-mutated lung cancer, global proteomics and phosphoproteomics data from cancer tissues were analyzed. Based on the non-inflamed phenotype of EGFR-driven non-small cell lung cancer (NSCLC), we found that targeting PKCδ is a promising strategy to induce tumor infiltrating lymphocytes (TIL). These turn “cold” tumors “hot” and make them more susceptible to ICB therapy. Our findings provide a novel avenue for enhancing the efficacy of tumor immunotherapy.

## Methods

### Materials

Rottlerin and PEP-005 were purchased from Sigma-Aldrich. CD3/28 antibody, IL-2, and fluorescence conjugated PD-L1/CD3/CD4/CD8 was obtained from Biolegend. Anti-PD-1 mAb (αPD-1) was purchased from BioXcell. CRISP-Cas9 plasmid of PKCδ was purchased from GenScript. RIPA buffer and primary antibodies against GAPDH, PD-L1, p-PKCδ, NF-κB, and p-P65 were purchased from Cell Signaling Technology. Total-PKCδ was obtained from Abcam. Anti-rabbit and anti-mouse secondary antibodies were purchased from Odyssey.

### Cell lines and culture

All cell lines were purchased from ATCC. Lung cancer cell lines H1975 (EGFR^L858R+T790M^ mutation), A549 (KRAS mutation), H460 (KRAS mutation), H1650 (EGFR^Exon19 del^), H820 (EGFR^L858R+T790M^ mutation), and H1819 (EGFR overexpression) were cultivated in RPMI 1640 medium supplemented with 10% fetal bovine serum, 100 U/ml penicillin, and 100 μg/ml streptomycin. Mouse cell lines LLC1 (mouse lung cancer cell) and NIH/3T3 (mouse embryonic fibroblast) cells were cultured in DMEM medium, while BEAS-2B (normal lung epithelial cell) was cultured in BEBM medium. PKCδ knock out cell line was generated in H1975 cells. EGFR^mut^ cells were generated by overexpressing EGFR^L858R+T790M^ in BEAS-2B cells. All the cells were cultivated at 37 °C in a 5% CO_2_ incubator. Agents (rottlerin and PEP-005) were dissolved in DMSO to generate the stock solutions (20 mM), and the stock solutions were diluted with full culture medium to their target concentrations.

### CRISPR-Cas9

CRISPR-Cas9 was used to knockout the target gene in NSCLC cells. Briefly, PRKCD CRISPR Guide RNA (Sequence: CTCCGCGGCGGTTCATCGTT) was constructed into lentivirus vector pLentiCRISPR v2 which was used as vehicle control. After transfected to target cells (H1975), puromycin was applied for screening the stable clone of PRKCD knockout.

### Real-time PCR

The expression level of mRNA was quantified by Real-time PCR by using FastStart Universal SYBR Green Master (Roche), following this protocol: 94 °C for 10 min, followed by 40 cycles at 94 °C for 10 s and 60 °C for 30 s. Actin was considered as internal standard. The primers sequences are as following:

ICAM1 Forward primer: TCTTCCTCGGCCTTCCCATA

ICAM1 Reverse primer: AGGTACCATGGCCCCAAATG

Actin Forward primer: GATATTGGCAACGACCCCCA

Actin Reverse primer: CCCAGCCAGGATCTTGAAGG

### Flow cytometry analysis of PD-L1 expression

After cells were trypsinized, 1 × 10^5^ cells were re-suspended in 100 mL of staining buffer containing 1μl APC-conjugated anti-human PD-L1 antibody (BioLegend) and incubated at room temperature for 15 min. After washing with PBS for 3 times, cells were analyzed by flow cytometry.

### T-cell killing assay

PKCδ^−/−^ H1975-GFP cells and the control were seeded in a 96-well plate overnight for adhesion. Human PBMCs were isolated from healthy donors by Ficoll centrifugation and immediately frozen in − 80°. PBMCs were activated with 2 μg/mL CD3/28 antibodies and 10 ng/mL IL-2, and then co-cultured with H1975 luciferase cells at 10:1 ratio. The cell number was calculated using an INCell Analyzer 6000 imaging system.

### Database acquisition

A large-scale and publicly available collection of multi-omic datasets from 103 lung adenocarcinoma (LUAD) cases in Chinese patients was released by the Tan Minjia group [11]. Integrative analysis of proteomic and phosphoproteomic data from this collection revealed cancer-associated characteristics in patients with EGFR mutations. The normalized iBAQ intensities released by Tan’s group were used in quantitative analysis of proteomic data. Downstream statistical analysis by Perseus software (https://www.perseus-framework.org/, version 1.5.5.3) was done following the standard protocol [12]. Samples were then grouped into NT and tumor group which was further divided into groups with or without EGFR mutation. Only proteins with 3 valid values in at least one group were kept. Student’s *T*-test was performed between experimental and control groups with or without EGFR mutation, with false discovery rate (FDR) < 0.05 and S0 = 0.1.

Next, the intensities of the phosphopeptide signals released by Tan’s group were used in quantitative analysis of phosphoproteomic data. Student’s *T*-test was operated first between tumor samples with and without EGFR mutation and, second, between experimental and control groups in EGFR mutation samples. Results with *p* < 0.05 were considered statistically significant. The missed proteins (zero or one value in the NT group, but five values in the tumor group) were rescued and combined with the phosphosites found to be significant.

### Proteomic analysis

One hundred μg of proteins were reduced and alkylated with 1mM dithiothreitol and 0.5mM iodoacetamide, respectively, followed by digestion with trypsin in 1:100 (w/w) ratio overnight. Then, peptides were desalted as described previously [13]. Finally, the desalted peptides were dried in vacuum and dissolved for LC-MS/MS analysis. LC-MS/MS analysis was performed on an Easy nLC system (Thermo Scientific, USA) coupled to a Q Exactive mass spectrometer (Thermo Scientific, USA) for 60 min. The mass spectrometer was operated in positive ion mode by full-scan MS scan (m/z 300-1550, resolution 70000) followed by data-dependent MS/MS scan (top 10 modes, resolution 17500). The MS data were analyzed using MaxQuant software (version 1.5.5.1) [14]. Proteins were identified against the human proteome sequences from UniProtKB (state July 2017, 70698 entries). 0.02 ppm and 7 Da were set for fragment ion and precursor ion tolerances, respectively. “2 missed cleavages” was enabled for tryptic peptide. Carbamidomethylation was chosen as static modification and oxidation and deamidation were selected as dynamic modifications. FDR of 0.01 was used in peptide identification. The label-free protein quantitation (LFQ) was performed using the LFQ algorithm [15]. Bioinformatics and statistical analyses were performed in Perseus software. Gene ontology enrichments were computed using the ‘enrichGO’ function from R package ‘clusterProfiler’ and top significantly enriched terms were selected [16].

### Protein−protein interaction analysis

The protein-protein relationship data was obtained from the STRING database based on experimental sources (version 11.0; https://string-db.org/). To find more novel EGFR interacting proteins, we also selected text mining resource in STRING analysis. Low-confidence edges (edges with a confidence score < 0.4) were removed from the network.

### Immunohistochemical staining (IHC) of human lung cancer tissue samples

One hundred patients’ samples were collected from West China Hospital following the hospital guidelines and patients signed informed consent in all cases. The slides with specimens were incubated with primary antibodies for PD-L1 and phosphor-PKC-δ (1:100 dilutions) overnight at 4 °C, which was detected by a biotin labeled anti-IgG secondary antibody and streptavidin-Horseradish peroxidase (HRP). Staining on the slides was quantified by colorimetric detection. According to the intensity of staining, specimens were classified into three levels: high (+++), medium (++), and low/negative (+/−).

### Tumor spheroid formation assay

NIH/3T3 cells and H1975 were trypsinized and collected. 2.5 × 10^4^ NIH/3T3 cells and 0.5 × 10^4^ H1975 cells were mixed and suspended in 200 μL culture medium in cell-repellent surface 96 well plates and centrifuged at 180g × 3 min. Plate was cultured overnight, and spheroid formation was observed.

### Cell surface marker and intracellular cytokines staining

Blood cells were collected by centrifuging at 350 g for 5 min. Cell pellets were re-suspended in 3 ml 1X RBC Lysis Buffer and incubated on ice for 5 min to lyse red blood cells. The lysate was then centrifuged for 5 min at 350 g, and supernatant was discarded. To prepare cells for cell surface marker staining, pre-incubation with Fc Receptor Blocking Solution was required, and then cells were added with conjugated fluorescent antibodies (e.g., anti-CD8-APC) on ice for 15–20 min in the dark. For some samples, further staining of intracellular cell components was required. These cells were fixed and permeabilized in Perm/Wash Buffer overnight. The fixed/permeabilized cells were then suspended in intracellular staining perm/wash Buffer with a conjugated antibody for 20 minu in the dark at room temperature. In the end, stained cells were loaded into a flow cytometer for analysis.

### Tumor dissociation, CD8^+^ and NK1.1^+^ isolation

The tumor tissues were dissociated, using a kit (Miltenyi Biotec), first by enzymatic digestion then gentleMACS™ Dissociators are used for mechanical dissociation steps. After dissociation, mixture was filtered using a 70-mm filter to obtain single-cell suspension. Next, CD8^+^ and NK1.1^+^ cells were isolated by manual magnetic labeling kit. Briefly, each 10^7^ total cells were incubated with 10 μL of Biotin-Antibody Cocktail and incubated for 5 min in 4 °C and then 20 μL of CD8^+^/NK1.1^+^ T Cell Micro-Bead Cocktail was mixed for 10 min. Finally, labeled cells were added to the column for isolation by magnetic MACS separator.

### Immune cell quantification in tumor spheroids

Three days after the tumor spheroids were seeded, PBMCs were added. PBMCs were isolated from healthy donors using the density gradient technique with the Ficoll PLUS from GE Healthcare. 5 × 10^4^ immune cells/well were added and co-cultured overnight. Before harvest, tumor spheroids were washed with PBS three times (1 minutes each time) to remove surface attached cells. After dissociation with Accumax (eBiosciences), the number of immune cells within the spheroids were quantified by flow cytometry.

### Xenograft mouse model

Animal studies were approved by the Ethical Committee of Macau University of Science and Technology. The mouse tumor model was established as previously described [17]. 1 × 10^6^ LLC1 mouse lung cancer cells/100 μl were subcutaneously injected into the right forelimb of C57BL/6 mouse. After 5 days, the mice with tumor volume reached 5 mm^3^ were divided into different treatment groups (*n* = 6): Control (treated with 200ul PBS /day by I.P), rottlerin (5mg/kg), anti-PD-1(200ug/time), and a combination treatment of rottlerin and anti-PD-1. Rottlerin was administered once a day and anti-PD-1 once a week by I.P administration. The tumor dimensions (length and width) were measured every 3 days, and the tumor volume was calculated by following equation: volume = (width^2^ × length)/2. Mice of each group were sacrificed at day 21 for taking images of tumor. For survival analysis, another six mice of each group were used for long-term study and calculated overall survival data.

### Statistical analysis

All data for the three experiments was analyzed using GraphPad Prism 5 (GraphPad Software, La Jolla, CA, USA). One way analysis of variance (ANOVA) was used to analyze the differences between three or more groups, and Student’s *t* test was used for comparisons of only two groups. Results were represented as means ± SEM. Any results with *p* < 0.05 were considered statistically significant.

## Results

### PKCδ served as the main downstream mediator in EGFR-mutated NSCLCs

**Fig. 1:**
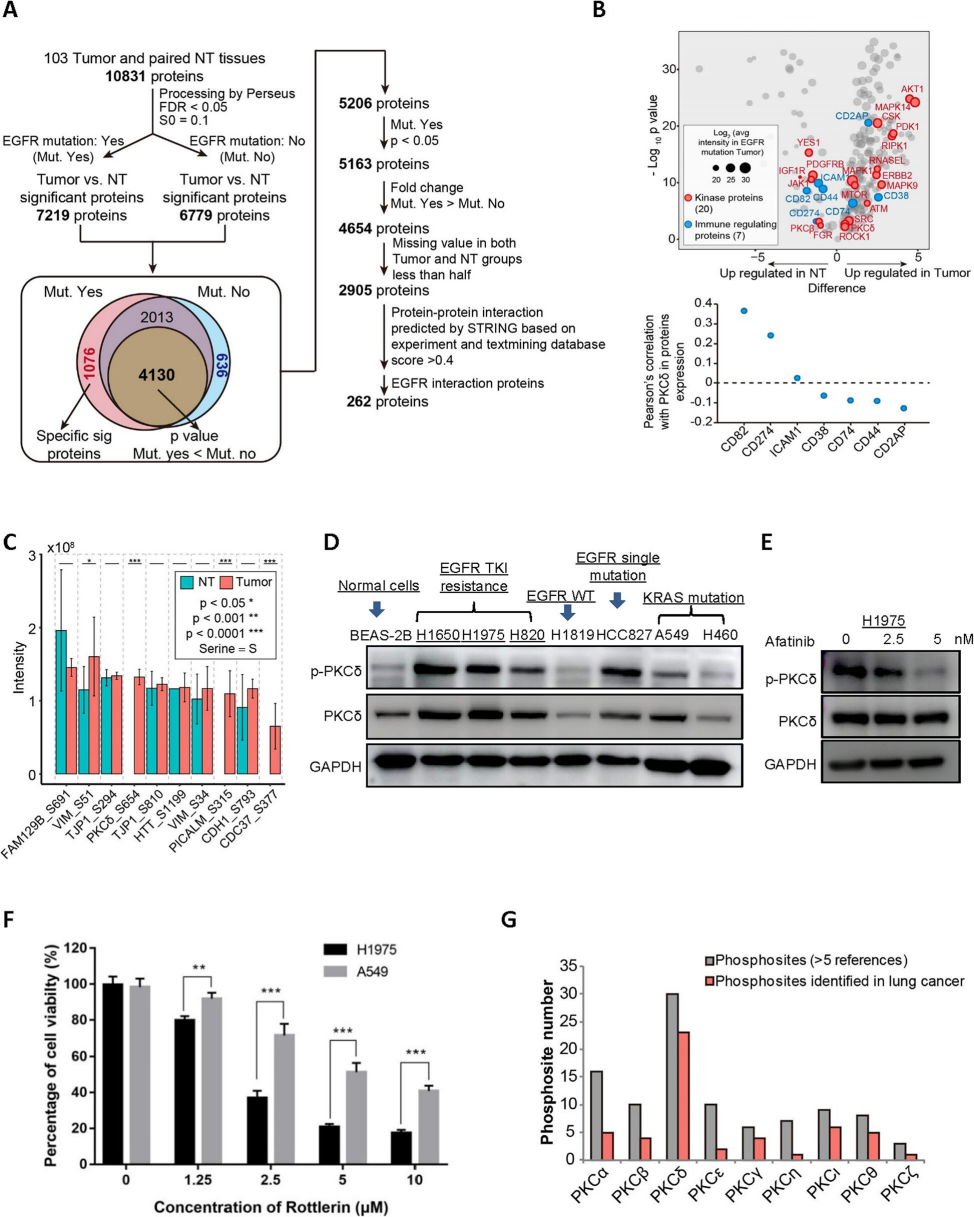
PKCδ is a key mediator of EGFR downstream signaling and closely associates with tumor non-inflamed phenotype. **A** Protein expression was comprehensively compared among normal tissue, wild-type, and mutated EGFR cancer. 7219 and 6779 significant proteins found in mutation and non-mutation groups respectively. **B** 262 proteins were found to be related to EGFR proteins, including 20 kinase proteins and 7 immune regulating proteins. **C** In phosphoproteomic analysis, 10 phosphosites of 8 proteins from these 262 EGFR related proteins were significantly regulated in EGFR mutation tumor. **D** In NSCLC, phosphorylation of PKCδ was directly related to EGFR active mutation. **E** Treatment of EGFR TKI suppressed the phosphorylation of PKCδ in H1975 cells. **F** Compared with non-EGFR mutated A549 cells, H1975 cells were more sensitive to the treatment of PKCδ inhibitor rottlerin. **G** Each PKC isoform contains 3–30 phosphorylation sites respectively and exhibits different profiling in lung cancer. The phosphorylation of PKCδ was most frequently correlated: more than 75% of its phosphorylation site was relatively increased

TME of EGFR-mutated NSCLCs displays immunological tolerance and a lack of T cell infiltration, which caused low clinical efficacy of immunotherapy [18–20]. To systematically analyzed the critical mediators associated with this immunosuppressive TME, proteomic and phosphoproteomic datasets from 103 lung adenocarcinoma (LUAD) cases [11], including 52 wild type (WT) and 51 EGFR-mutated tumors, and paired normal tissues (NT) were selected (Fig. 1A). Compared with NT, 7219 and 6779 significantly changed proteins were found in EGFR mutated and WT groups respectively. Among them, 1076 specific proteins were exclusively identified in EGFR-mutated group and 4130 proteins with a lower adjust *p* value were found in mutated group as well when compared with WT group. Of these, 2905 proteins were considered high confidence, based on adjusted *p* value < 0.05. The interactions of these proteins were then characterized by the STRING analysis: 262 proteins were finally identified to relate with EGFR signaling (Fig. 1A), which included 20 kinases and 7 immune regulating proteins (Fig. 1B).

Next, the 20 protein kinase candidates which are targetable for clinical use from these 262 EGFR related proteins were selected for further phosphoproteomic analysis. Ten phosphosites of 8 protein kinase candidates were significantly regulated in EGFR-mutated tumor (Fig. 1C). It was noted that phosphorylation of PKCδ, PICALM, and CDC37 were specifically enhanced in the tumor group, but few were identified in NT group. Among these three proteins, only PKCδ is directly involved in immune responses [21], while PICALM is a clathrin assemble protein [22], and CDC37 is a chaperone directing Hsp90 to its target kinases [23]. Given these, we hypothesized that PKCδ serves as the critical mediator for inducing immunosuppressive TME in EGFR-mutated lung cancer.

The correlation between PKCδ and EGFR was ascertained in different NSCLC cell lines. As shown in Fig. 1D, the expression level of both total and phosphorylated PKCδ was remarkably enhanced in EGFR-mutated cells, which is consistent with the results of proteomics. We then treated EGFR-mutated H1975 cells with EGFR TKI afatinib and the phosphorylation of PKCδ in H1975 was significantly suppressed (Fig. 1E). In addition, rottlerin (inhibitor of PKCδ) was shown to be more effective in inhibiting the growth of H1975, when compared with non-EGFR mutated NSCLC cells (Fig. 1F). These results further supported that PKCδ is the main downstream of EGFR.

To corroborate the specific function of PKCδ in lung cancer, we utilized PhosphoSitePlus, a database dedicated to mammalian post-translational modifications, to comprehensively investigate the phosphorylation of different PKC isoforms in lung cancer. Each PKC isoform contains 3−30 phosphorylation sites respectively and exhibits different profiling in lung cancer (Fig. 1G and Fig S1). Notably, the phosphorylation of PKCδ was most frequently correlated: more than 75% of its phosphorylation site was relatively increased. These further supported the importance of activation of PKCδ in lung tumorigenesis.

### Targeting PKCδ enhanced intratumoral T cells diversity and inhibited tumor growth

**Fig. 2:**
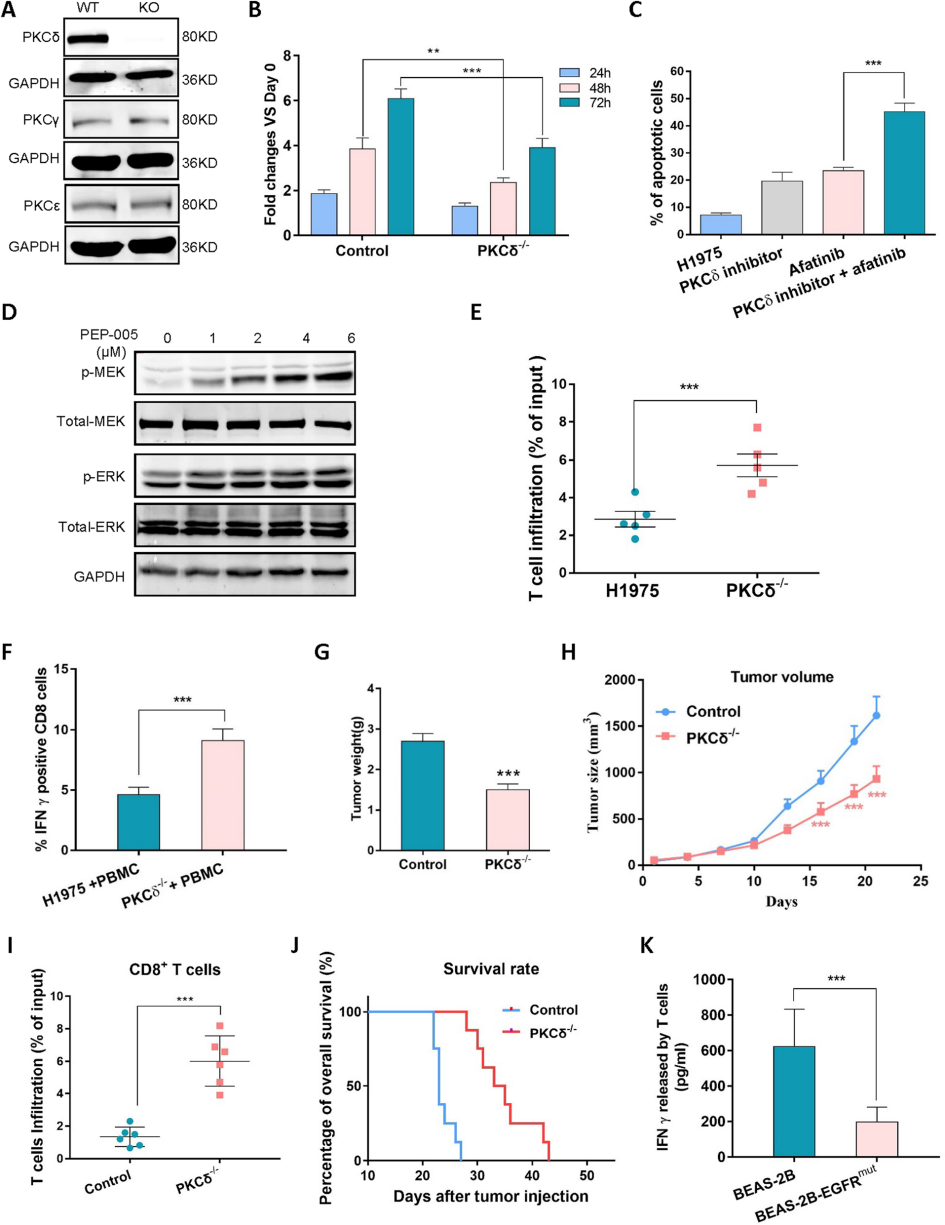
Targeting PKCδ inhibited the growth of tumor and enhanced intratumoral T cell diversity. **A** PKCδ^−/−^ cell model based on EGFR double mutated cell H1975. **B** MTT assay showed that compared with control group, PKCδ depletion remarkably suppressed growth of cancer cells. **C** Flow cytometry analysis demonstrated that combination of PKCδ inhibitor and EGFR TKI significantly enhanced apoptotic percentage of resistant cancer cells. **D** PKCδ activator PEP-005 activated PI3K-ERK signaling. **E**, **F** Flow cytometry results showed that knock out of PKCδ promoted infiltration of T cells into TME and increased release of cytokine IFN-γ from CD8^+^ T cells. **G**, **H** In vivo EGFR-mutated xenograft nude mice model, PKCδ^−/−^ remarkably decreased tumor weight and size. **I**, **J** After knockout PKCδ, the number of TILs detected by flow cytometer was increased significantly in TME and survival time of mice was prolonged. **K** ELISA assay proved that compared with control (normal lung epithelial cells, BEAS-2B), co-cultured with BEAS-2B overexpressed mutated EGFR^L858R+T790M^, T cells activity was compromised and thus the secretion of IFN-γ was suppressed. Data was triplicated and represented as mean ± SEM (*p < 0.05, **p < 0.01, ***p < 0.001)

To validate the potential roles of PKCδ in lung tumorigenesis, we firstly knocked out PKCδ on H1975 cells (Fig. 2A). As a result, PKCδ^−/−^ significantly compromised the growth of H1975 cells, suggesting that PKCδ is required for EGFR-mutant NSCLC cells (Fig. 2B). Meanwhile, since PKCδ serves as the main downstream signaling molecule of EGFR, rottlerin was used to overcome the EGFR TKI resistance. As shown in Fig. 2C and Fig. S2A, the combination of rottlerin and afatinib significantly increased the proportion of TKI-resistant cancer cells that became apoptotic. On the contrary, PKCδ activator PEP-005 was used to treat EGFR WT NSCLC H460 cells. Its application resulted in the activation of PI3K-ERK signaling, the main pathway related to EGFR induced cell proliferation (Fig. 2D). Altogether, the evidence builds a strong case that PKCδ is the main mediator of EGFR mutation.

Next, to determine whether tumor PKCδ affects the filtration of T cells to TME, tumor spheroids of H1975 (with or without PKCδ^−/−^) were established to mimic human solid tumor in vitro. Compared with the control, PKCδ^−/−^ in H1975 significantly promoted the infiltration of T cells into the TME (Fig. 2E). Meanwhile, in T cell killing assay, when compared with cultured with H1975, the cytotoxicity of T cells was much higher in PKCδ^−/−^ H1975 group: the generation of IFN-γ of CD8^+^ T cells were significantly enhanced (Fig. 2F). These indicated that PKCδ is responsible for immunosuppressive effect on cancer.

The immune suppressive effect of tumor PKCδ was examined in vivo as well. Lung carcinoma xenograft models (H1975 cells with or without tumor PKCδ^−/−^) were established in nude mice and administrated with human PBMC by tail vein injection. As expected, the tumor weight and size of PKCδ^−/−^ group were remarkably decreased (Fig. 2G, H), while the frequency of TILs (Fig. 2I) and survival time of animals (Fig. 2J) were increased significantly. Moreover, we overexpressed mutated EGFR^L858R+T790M^ in normal lung epithelial cells (BEAS-2B), to strengthen the case for EGFR activating PKCδ and test their role in regulating T cell activation. As expected, overexpression of mutated EGFR suppressed the secretion of IFN-γ in a co-culture model, which indicated the lower T cell activity (Fig. 2K). These results suggested that deletion of tumor PKCδ contributes to enhancing intratumoral T cells diversity and inhibiting tumor growth.

### PKCδ established a TME physical barrier and suppressed T-cell infiltration by activating NF-κB/ICAM1 signaling

**Fig. 3:**
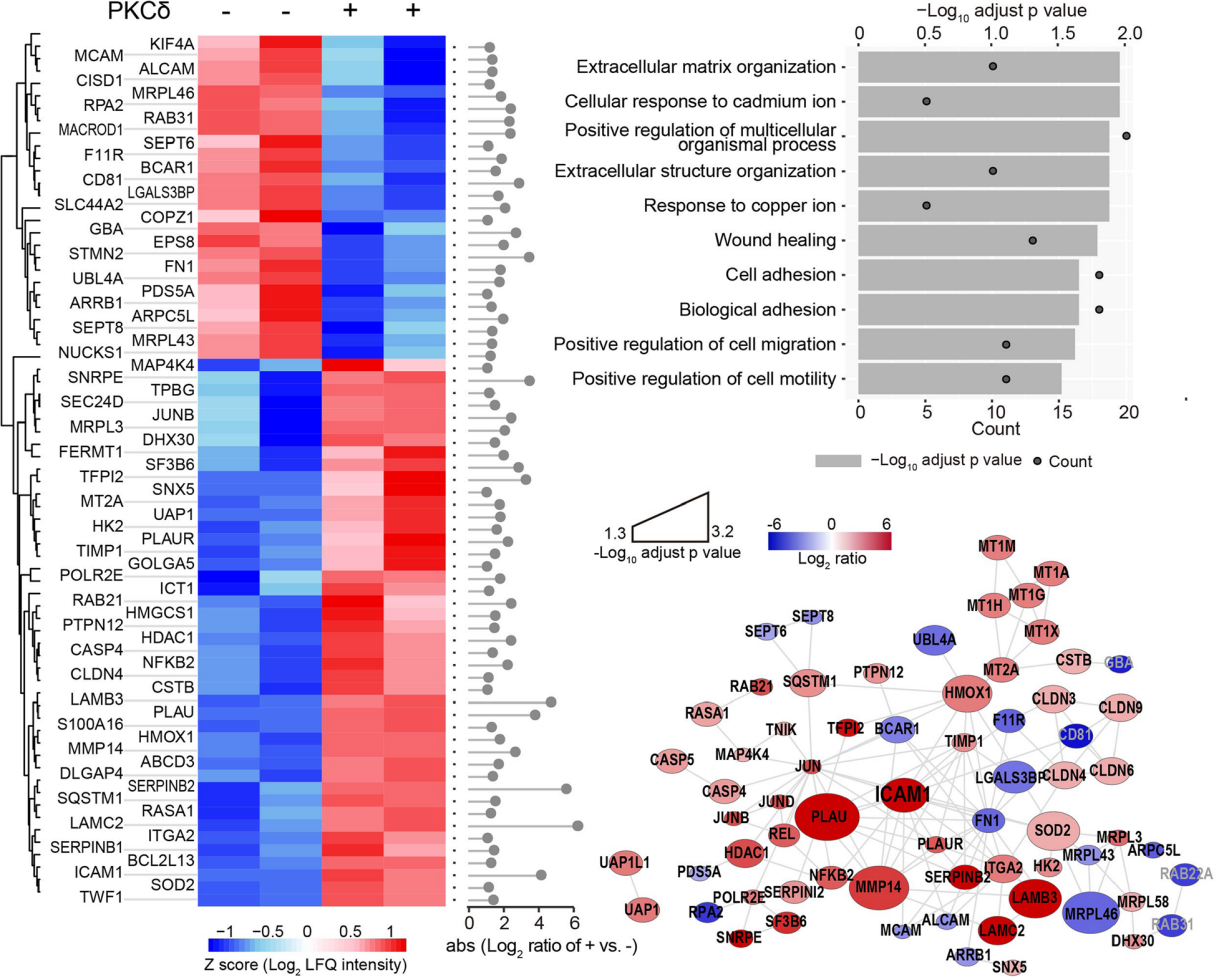
Proteomics analysis demonstrated that PKCδ promotes establishment of physical barrier in TME and suppresses T-cell trafficking by inducing NF-κB/ICAM1 signaling. **A** Proteomics results showed that 70 significantly changed protein groups from two clusters between H1975 with or without PKCδ^−/−^ were identified. The absolute transformed ratio was noted on the right. **B** Top 10 enriched terms of Gene Ontology biological processes were annotated from these differentially expressed proteins. **C** Protein–protein interaction network was constructed from these differentially expressed proteins in STRING. ICAM1 and its connected proteins were considered as the center of signal network

To investigate how PKCδ reduced the infiltration of lymphocytes in TME, we performed a proteomic analysis to compare PKCδ WT H1975 cells with PKCδ^−/−^ ones. To model proteome changes, we integrated co-expression clustering analysis, protein expression fold change, Gene Ontology analysis, and the protein–protein interaction (PPI) network. Significant protein profiling (fold change ≥ 2 and *p* value < 0.05) was clustered into two groups (Fig. 3A). In these clusters, 44 protein groups were significantly upregulated, and 26 protein groups were downregulated in PKCδ^−/−^ cells, in which top 5 most differentially expressed proteins were LAMC2, SERPINB2, LAMB3, ICAM1, and PLAU. The biological process (BP) of the differentially expressed proteins was annotated by Gene Ontology analysis. The top 10 enriched terms were listed in Fig. 3B, including multicellular organismal process and cell/biological adhesion with the highest number of protein counts. To obtain a comprehensive view of their interaction, we performed STRING protein–protein interaction network analysis. The analysis showed that these significant proteins are highly connected in the same signaling network (Fig. 3C).

By clustering analysis of these differential proteins, the nuclear factor kappa B (NF-κB) and cell-cell adhesion signaling pathways were identified as the two most significantly changed pathways (Fig. 3B, C). Targeting NF-κB signaling was demonstrated as an effective therapeutic approach against cancer [24, 25]. The cell-cell adhesion signaling pathway is responsible for tumor invasion and metastasis by regulating a series of genes including matrix metallopeptidase (MMP), integrin subunit alpha (ITGA), and immunoglobulin-like cell adhesion molecule 1 (ICAM1) [26]. Meanwhile, as reported, NF-κB is upstream of the cell-cell adhesion associated gene, and thus we suspected that NF-κB and its downstream signaling are responsible for EGFR-mutation caused T cell non-inflamed characteristics. To confirm this, we firstly used PKCδ inhibitor to determine its correlation with NF-κB signaling. As shown in Fig. S2B and S2C, activity of NF-κB and expression of ICAM1 were significantly inhibited by rottlerin. This indicated that PKCδ suppresses T-cell trafficking to tumors by activating NF-κB/ICAM1 signaling.

### PKCδ enhanced PD-L1 expression and induced immune escape

**Fig. 4:**
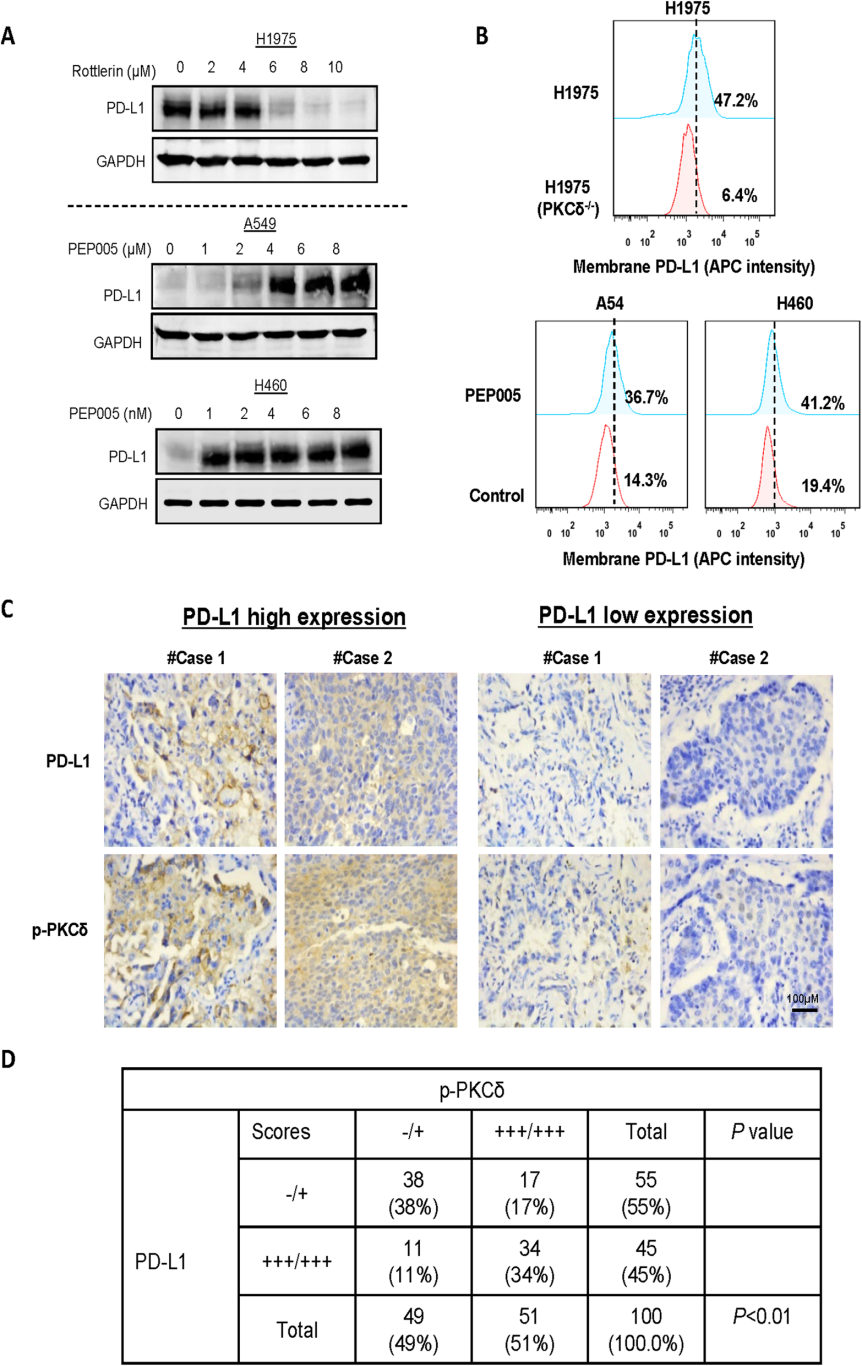
PKCδ enhanced PD-L1 expression and induced immune escape. **A** PKCδ inhibitor rottlerin significantly suppressed the expression of PD-L1 in EGFR-mutated H1975, while PKCδ activator PEP-005 increased PD-L1 in EGFR-WT A549 and H460 cells. **B** FACS results showed that PKCδ knockout in H1975 decreased cell surface PD-L1 level, whereas PEP-005 upregulated its expression in A549 and H460. **C**, **D** High phosphorylation of PKCδ was detected in 51.0% of 100 lung tumor specimens, 34 cases out of 51 (66.7%) showed high PD-L1 expression (−/+ represents that that both the expression of p-PKCδ and PD-L1 are low or even non-expression, while +++/+++ means that both the expression of p-PKCδ and PD-L1 are high)

As mentioned in Fig. 1B, PKCδ was reported to be associated with 7 immune-regulating proteins, CD274 (PD-L1) was a close correlation with PKCδ in protein level. We further tested whether PKCδ can regulate the expression of PD-L1. As shown in Fig. 4A, PKCδ inhibitor rottlerin and activator PEP-005 were used to treat different lung cancer cells. Rottlerin effectively suppressed the expression of PD-L1 in H1975 and PEP-005 remarkably enhanced PD-L1 expression in KRAS mutated A549 and H460 cells. Since PD-L1 is a cell surface receptor that interacts with PD-1 to transduce inhibitory signals to immune cells, its cell surface level was determined by FACS: corresponding data with similar changes of total PD-L1 was acquired (Fig. 4B). Next, the correlation between the phosphorylation of PKCδ and PD-L1 expression was further validated in human lung tumor specimens by immunohistochemistry (IHC). High phosphorylation of PKCδ was detected in 51.0% of the 100 lung tumor specimens (Table S1), of which 34 cases (66.7%) showed high PD-L1 expression (Fig. 4C, D). Pearson’s chi-square (*χ*^2^) test was applied, and the result showed a positive correlation exists between these two proteins (Fig. 4D). These data indicate that activation PKCδ promotes PD-L1 expression in lung cancer, which induces immune escape and contributes to tumor growth.

### Suppressing PKCδ enhanced the efficacy of αPD-1 in vivo

**Fig. 5:**
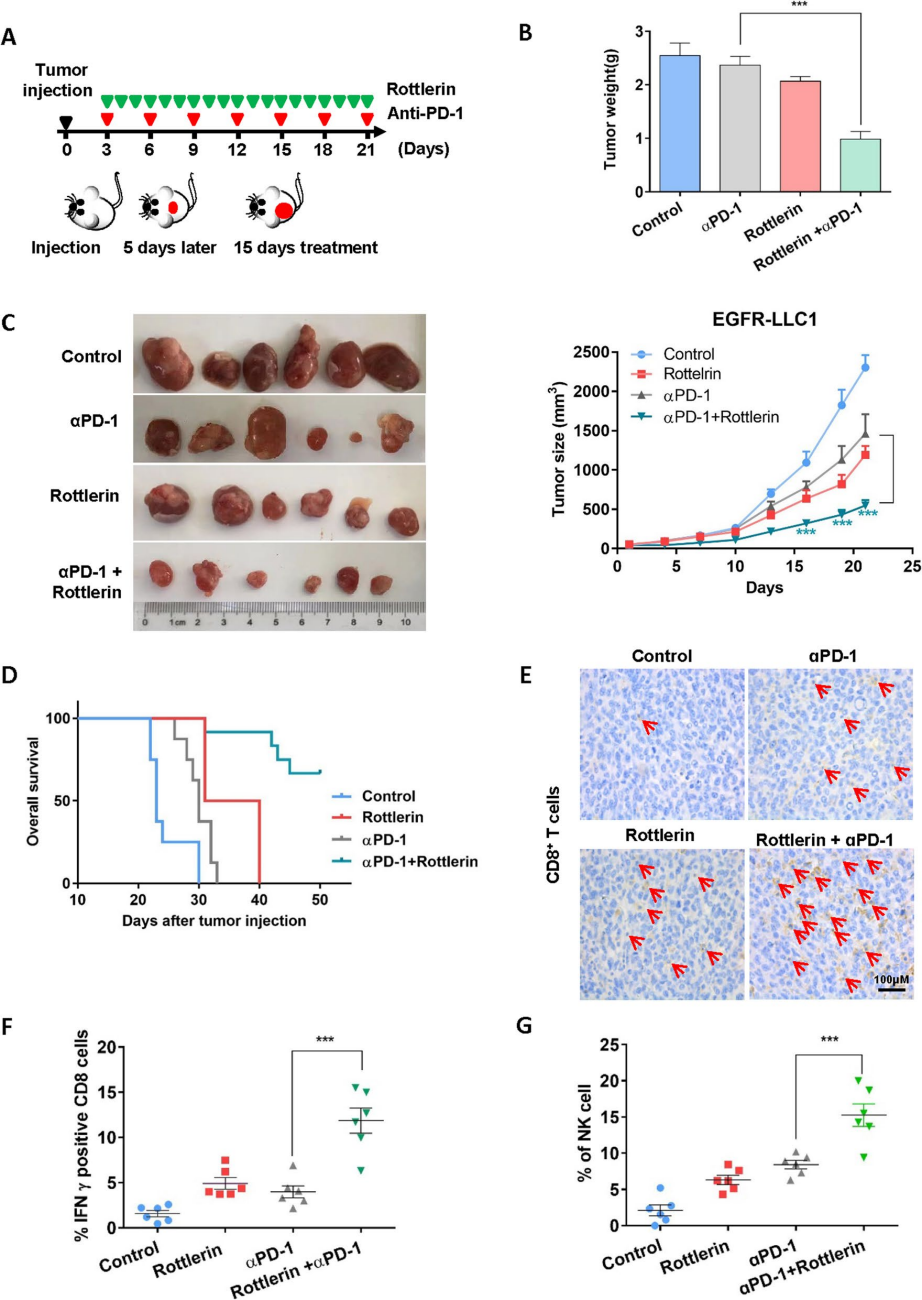
Suppressing PKCδ enhanced the efficacy of αPD-1 in vivo mouse model. **A** Combinational strategy of ɑPD-1 and rottlerin in vivo. **B**, **C** In EGFR-mutated mouse lung cancer model, tumor volume and weight were largely limited by combinational treatment of ɑPD-1 and rottlerin. **D** The overall survival time of combined group was much longer than that of control or single treatment groups. **E** IHC staining assay showed that blocking PD-1/PD-L1 signaling, PKCδ inhibitor remarkably enhanced number and activity of tumor infiltrating CD8^+^ T cells. **F** Flow cytometry analysis demonstrated that the expression of IFN-γ in CD8^+^ T cells and **G** the number of NK cells were increased by combined treatment. Data was triplicated and represented as mean ± SEM (*p < 0.05, **p < 0.01, ***p < 0.001)

The above findings supported a novel mechanism to remodel non-inflamed tumors to inflamed ones which are sensitized to ICB therapy. To validate the importance of the findings, the efficacy of rottlerin and αPD-1 single or combinational treatments were compared in EGFR-LLC1 mouse xenograft model. Therapeutic efficacy was evaluated by three indicators: (i) tumor volume and weight, (ii) mouse survival time, and (iii) the number and activation of TILs within the TME. The therapeutic strategy was shown in Fig. 5A. Combinational treatment greatly limited tumor growth and its efficacy were better than in single treatment groups. On day 21, the tumor volume and weight of combinational therapeutic group were approximately 1/4 of the control (Fig. 5B, C). The survival time of the combined treatment group was also much longer than that of the control or the single treatment groups (Fig. 5D). Moreover, together with αPD-1, PKCδ inhibitor remarkably enhanced the number and activity of tumor infiltrating CD8^+^ T cells (Fig. 5E). With IFN-γ taken as an indicator of T cell activation, staining in tumor biopsies was increased from 3 % in the control group to nearly 20.0% in the combined therapeutic group (Fig. 5F and Fig. S2D). The number of natural killer (NK) cells was also increased in the combined group (Fig. 5G and Fig. S2E). Taken together, the results suggested that inhibiting PKCδ is an effective way to enhance the efficacy of αPD-1 in EGFR-mutated lung cancer.

## Discussion

Infiltration of functional cytotoxic T lymphocytes into the TME is essential for inducing durable clinical responses to ICB therapy, and the presence of sufficient TILs is a critical indicator of good prognosis for patients [27]. In other words, ICB works best against so-called “hot” tumors (T cell inflamed tumors). These resulted in which only a minority of cancer patients can recruit sufficient TILs in established tumors and merely 20–30% clinical response rate of ICB. Patients with EGFR mutations especially exhibited uninflamed phenotypes and weak immunogenicity and consequently showed an unfavorable response to PD-1 blockade immunotherapy [18, 28]. Development of an effective way to facilitate T cell infiltration into the TME is urgently required for current clinical therapy.

In this study, we found that PKCδ is responsible for the non-inflamed phenotype of EGFR-mutated tumors. PKCδ activates NF-κB and mediates the upregulation of cell-cell adhesion genes, which, in turn, results in the formation of a physical barrier, decreasing T cell infiltration into the TME and ultimately failure of ICB therapy. The finding was further verified by combining treatment of PKCδ inhibition and αPD-1, resulting in significantly enhanced antitumor efficacy of αPD-1. Therefore, our study provides insight into overcoming the lack of effective strategies to enhance the clinical efficacy of ICB therapy.

Recently, PKC isozymes are demonstrated to be closely involved in multiple signal transduction systems that respond to a variety of external signals, including hormones, growth factors, and other membrane receptor ligands [29, 30]. Meanwhile, PKC isozymes are also widely involved in tumorigeneses. For example, PKCι/λ and PKCζ, are now considered fundamental regulators of tumorigenesis [31]; PKCε acts as a key regulator of mitochondrial redox homeostasis in acute myeloid leukemia [32]. Among different isoforms, PKCδ was reported as a critical regulator of immune homeostasis and closely involved in autoimmune disease and cancer progression [21, 33]. The oncogenic role of PKCδ has been demonstrated in preclinical and clinical data, including promotion of lung KRAS-dependent tumorigenesis [34] and negative correlation with the prognosis of ErbB2-driven tumorigenesis [35]. Interestingly, ectopic expression of PKCδ in NSCLC was shown to lead to TKI-resistance in EGFR-mutant lung cancer patients [36]. This resistance is typical of “cold” tumors. This previous research is consistent with our findings and further supports the important role of PKCδ in inducing an immunosuppressive TME.

As a signaling molecule downstream of PKCδ, NF-κB family members and their regulated genes have been linked to malignant transformation, tumor cell proliferation, survival, angiogenesis, invasion/metastasis, and therapeutic resistance. NF-κB is reported to be closely involved in cancer initiation and progression [37, 38]. The concept of “NF-κB addiction” was widely accepted in cancer [39]. It was reported to constitutively activate in different types of human cancers and regulate various oncogenic genes in cancer development and progression. Moreover, the association of the immunosuppressive TME with NF-κB has been proven in many cancers. For example, human ovary cancer constitutively activates NF-κB signaling and produces cytokines which impair T cell activity and promote expansion of immunosuppressive MDSCs [40]. Although the pro-inflammatory effect of NF-κB has been well established, based on this research, for certain types of tumors, temporary blocking of NF-κB signaling will contribute to enhancing clinical efficacy of cancer immunotherapy.

Induction of PD-L1 is another important mechanism of PKCδ promoting cancer progress and escaping immune surveillance, which also uncovers a novel pathway between EGFR and PD-L1. Especially for TKI resistant NSCLC, it provides a potential explanation why such tumors are prone to the uninflamed status in TME.

## Conclusions

Our study proposes a potential mechanism which enhanced the efficacy of cancer immunotherapy in lung cancer. PKCδ as a common mediator of EGFR-mutated lung cancer induces an immunosuppressive effect on cancer. Consequently, targeting PKCδ could increase the response rate to PD-1/PD-L1 blockade, especially in non-inflamed tumors.

## Supplementary Information

**Additional file 1: Figure S1.** Each PKC isoform contains 3-30 phosphorylation sites respectively and exhibits different profiling in lung cancer. The phosphorylation of PRKCD/ PKCδ was mostly correlated with lung cancer. **Figure S2.** The combination of rottlerin and afatinib significantly increased the proportion of TKI resistant cancer cells that became apoptotic. (B) Activity of NF-κB and expression of ICAM1 were significantly inhibited by rottlerin. (C) Rottlerin significantly inhibited the RNA expression level of ICAM1. (D and E) Results of flow cytometer detection showed that IFN-γ of CD8^+^ T cells and the number of NK cells from TME were increased in combined treatment. Data was triplicated and represented as mean ± SEM (*p < 0.05, **p < 0.01, ***p < 0.001).

**Additional file 2: Table S1.** The clinical information of 100 patients’ samples.

## Data Availability

All data are available in the main text.

## Abbreviations

NSCLC: Non-small cell lung cancer
PBMCs: Peripheral blood mononuclear cells
PKC: Protein kinase C
TIL: Tumor infiltrating lymphocytes
TME: Tumor microenvironment
