# Supplementary figures and images for: Tumor PKCδ instigates immune exclusion in EGFR-mutated non–small cell lung cancer
Source: BMC Med. 2022 Dec 8;20:470. doi: 10.1186/s12916-022-02670-0 (PMC9733210; doi:10.1186/s12916-022-02670-0)

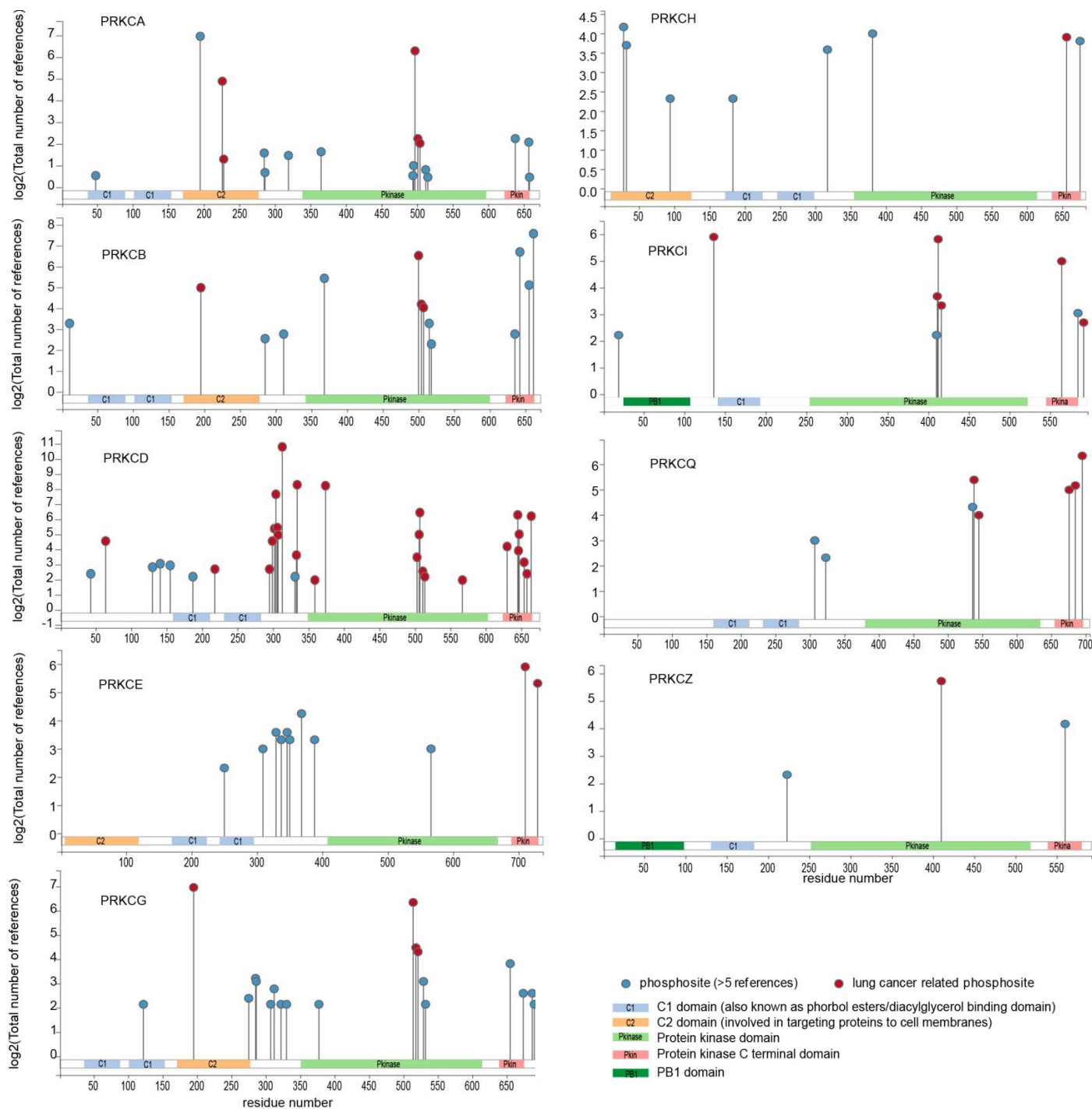

A

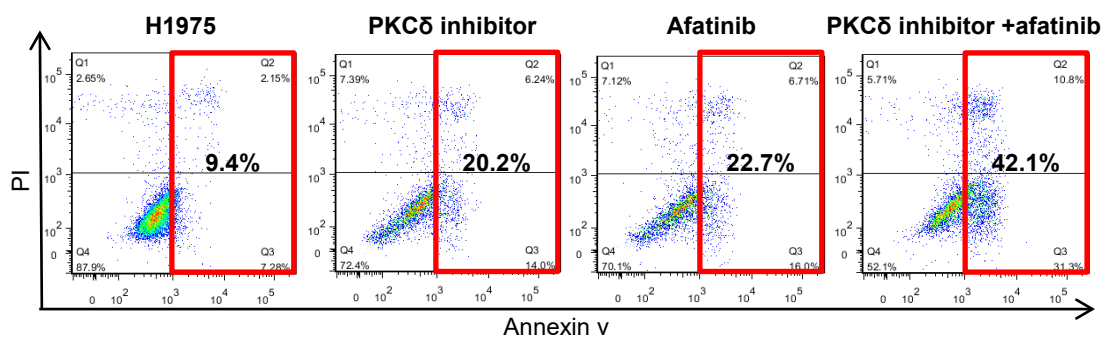

B

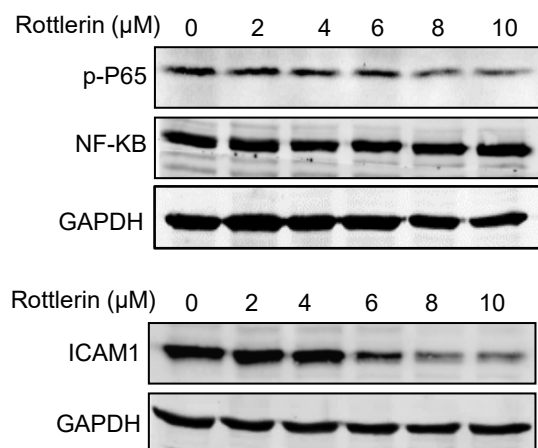

C

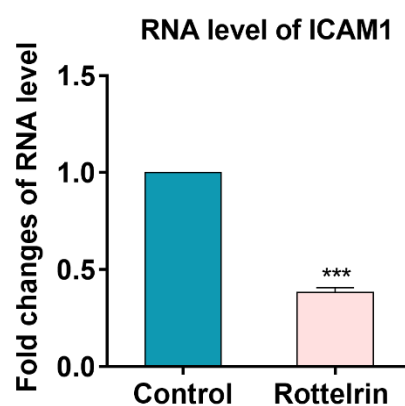

D

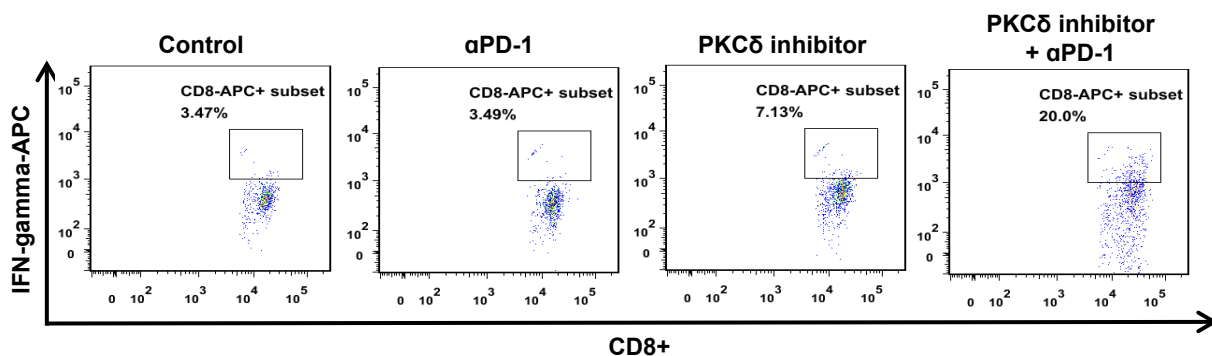

E

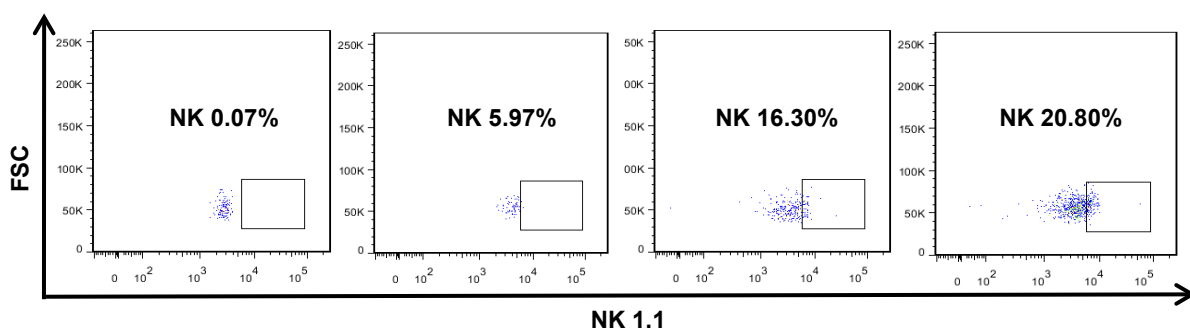

Supplement: Supplementary file 1 — Additional file 1: Figure S1. Each PKC isoform contains 3-30 phosphorylation sites respectively and exhibits different profiling in lung cancer. The phosphorylation of PRKCD/ PKCδ was mostly correlated with lung cancer. Figure S2. The combination of rottlerin and afatinib significantly increased the proportion of TKI resistant cancer cells that became apoptotic. (B) Activity of NF-κB and expression of ICAM1 were significantly inhibited by rottlerin. (C) Rottlerin significantly inhibited the RNA expression level of ICAM1. (D and E) Results of flow cytometer detection showed that IFN-γ of CD8+ T cells and the number of NK cells from TME were increased in combined treatment. Data was triplicated and represented as mean ± SEM (*p < 0.05, **p < 0.01, ***p < 0.001). [file 12916_2022_2670_MOESM1_ESM.pdf]
